# Diagnostic Utility of Direct Immunofluorescence on Paraffin-Embedded Skin Biopsy Samples for the Diagnosis of Autoimmune Vesiculobullous Lesions

**DOI:** 10.7759/cureus.56916

**Published:** 2024-03-25

**Authors:** Praveen BK, Hemlata Panwar, Deepti Joshi, Dinesh Asati, Jai K Chaurasia, Dega Vamseekrishna, Bertha A Rathinam, Neelkamal Kapoor

**Affiliations:** 1 Department of Pathology and Laboratory Medicine, All India Institute of Medical Sciences, Bhopal, Bhopal, IND; 2 Department of Dermatology, STD and Leprosy, All India Institute of Medical Sciences, Bhopal, Bhopal, IND; 3 Department of Anatomy, All India Institute of Medical Sciences, Bhopal, Bhopal, IND

**Keywords:** immunoreactants, direct immunofluorescence, pemphigus, paraffin-embedded tissue, autoimmune bullous

## Abstract

Background

Autoimmune vesiculobullous diseases (AIBDs) are a group of diseases characterized by blisters of the skin/mucosa due to the presence of circulating autoantibodies against antigens in the epidermis or the dermo-epidermal junction. Direct immunofluorescence (DIF) for immunoglobulin (Ig)G, IgC3, and IgA on fresh-frozen tissue is the gold standard diagnostic test for AIBDs. However, DIF in the absence of frozen tissue is challenging for the diagnosis of AIBDs. This study aimed to analyze the practical utility of DIF using paraffin-embedded skin biopsy rather than fresh frozen tissue for the diagnosis of AIBDs.

Methodology

This cross-sectional comparative study included 30 cases of AIBDs. DIF for IgG and IgA was performed on paraffin-embedded tissue (PE-DIF) after proteinase digestion on histopathologically confirmed 15 pemphigus vulgaris (PV), three pemphigus foliaceous (PF), four bullous pemphigoid (BP), three dermatitis herpetiformis (DH), three subcorneal pustular dermatosis (SCPD), and one case each of linear IgA disease and pemphigoid gestationis (PG). PE-DIF staining pattern was compared with the DIF on fresh frozen tissue (FF-DIF).

Results

All cases of PV and PF showed an intercellular IgG chicken wire staining pattern similar to FF-DIF. However, background staining was more intense in PV cases while less intense in PF cases. Three BP cases showed linear IgG staining in PE-DIF. DH, SCPD, linear IgA disease, and PG cases did not show IgG positivity. Out of three DH cases, two cases showed granular IgA positivity while linear IgA positivity along the basement membrane was seen in a single case of linear IgA disease. Negative IgG staining was observed in SCPD. Immunofluorescence in PE-DIF was rapidly deteriorating than in FF-DIF.

Conclusions

DIF done on paraffin-embedded tissue can be used as a supplement and salvage technique with histopathology for the diagnosis of AIBDs, particularly when a cryostat facility for frozen tissue is not available and the patient is unable to undergo a second biopsy procedure.

## Introduction

Autoimmune vesiculobullous diseases (AIBDs) form a heterogeneous spectrum of dermatological conditions marked by the formation of blisters and erosions on the skin and mucosal surfaces [1]. The underlying pathology involves circulating autoantibodies targeting specific antigens within the epidermis or dermo-epidermal junction [2]. There is the presence of cytolysis, spongiosis, reticular degeneration, acantholysis, and basement membrane zone (BMZ) destruction associated with blister formation [3].

This diverse group includes pemphigus vulgaris (PV), pemphigus foliaceous (PF), paraneoplastic pemphigus, bullous pemphigoid (BP), cicatricial pemphigoid, dermatitis herpetiformis (DH), epidermolysis bullosa simplex, and linear immunoglobulin (Ig)A dermatosis, each presenting with distinct clinical and immunological features necessitating meticulous evaluation [4].

On histopathology, pemphigus is identified by the presence of an intraepidermal blister and immunopathologically by in vivo bound as well as circulating IgG antibodies which are directed against desmosomal adhesion proteins present on the surface of keratinocytes. The cardinal target antigens involved in pemphigus include desmogleins (Dsg), such as Dsg 1 and 3. These are found in the skin and mucosa [5]. The most common type of tissue‐bound antibodies in pemphigus are IgG, which may be demonstrated in both direct and indirect immunofluorescence studies [6]. Further, IgM, IgA, and IgE antibodies may also be seen at times. The location and deposition pattern of immunoreactants contribute to the classification of several types of immune-mediated disorders [7].

Diagnosing vesiculobullous lesions requires a comprehensive, multidisciplinary approach encompassing clinical, histopathological, and immunological assessments. Clinical examination involves scrutinizing the skin and mucosal surfaces and mapping the location, distribution, and morphology of blisters. A detailed patient history aids in narrowing the differential diagnosis, considering factors such as onset, progression, and associated symptoms.

Histopathological examination of lesional skin serves as a cornerstone, providing microscopic insights into blister formation, inflammatory infiltrates, and epidermal/dermal changes. Immunological evaluation, crucial for confirmation, traditionally relies on direct immunofluorescence (DIF) on fresh frozen tissue. However, the technical challenges and resource limitations associated with fresh frozen tissue underscore the quest for alternative approaches. Recent strides in diagnostic techniques have addressed these challenges, with emerging technologies such as enzyme-linked immunosorbent assay, immunoblotting, and multiplex immunoassays showing promise [8]. The protocol promises accessibility and reliability, utilizing readily available paraffin-embedded tissue sections.

The objectives of this study include assessing the reproducibility of immunofluorescence staining from the devised protocol and determining the feasibility and accuracy of paraffin-embedded biopsies for DIF in diagnosing AIBDs.

## Materials and methods

This cross-sectional comparative study conducted at a tertiary care hospital aimed to assess the practical applicability of DIF on paraffin-embedded skin biopsy specimens for diagnosing AIBDs. Ethical guidelines were strictly followed and informed consent was obtained. Institutional review board approval was secured from the Institutional Human Ethics Committee-Post Graduate Research (approval number: IHECPGRMD040). A total of 30 cases were included, comprising both newly diagnosed and partially treated individuals. Exclusion criteria encompassed patients with infectious bullous lesions (viral, fungal, and bacterial), various vesiculobullous disorders (drug-induced, metabolic, pustular psoriasis, erythema toxic neonatorum, transient neonatal pustular melanosis, friction blisters, allergic and irritant contact dermatitis, phototoxic, and photoallergic reactions), and those lacking definitive bullae for proper histopathological evaluation under light microscopy. This stringent selection process ensured a focused investigation into the diagnostic utility of DIF in AIBDs.

Initially, during the standardization and validation of the DIF-P method, we applied this technique on the normal skin tissue for negative controls. After satisfactory negative results, we restricted to diagnosed cases of immunobullous lesions for the validation and standardization of the DIF-P method. Skin biopsies from intact vesicles and adjacent normal tissue were fixed in 10% buffered formalin for routine histological analysis and subjected to paraffin-embedded direct immunofluorescence (PE-DIF).

Manual PE-DIF was done using a protocol devised from the available literature and subsequent standardizations The PE-DIF protocol was meticulous. Paraffin-embedded blocks were cut into 3-4 µm thick sections and underwent heat fixing at 60°C for 30 minutes to ensure adhesion. Deparaffinization was done, and after rinsing with distilled water, Tris buffer treatment (pH 9.0) was done for 15 minutes to prepare the tissue for immunofluorescence staining. To enhance antigen retrieval, proteinase-K (75 μL) was applied, and enzymatic digestion occurred at 37°C for 10 minutes. This step exposed target antigens for subsequent antibody binding. Sections were washed with Tris buffer at 4°C for 15 minutes, followed by rinsing with phosphate-buffered saline (PBS, pH 7.4) to remove excess proteinase-K, preparing sections for antibody incubation.

Immunofluorescence staining

**Figure 1:**
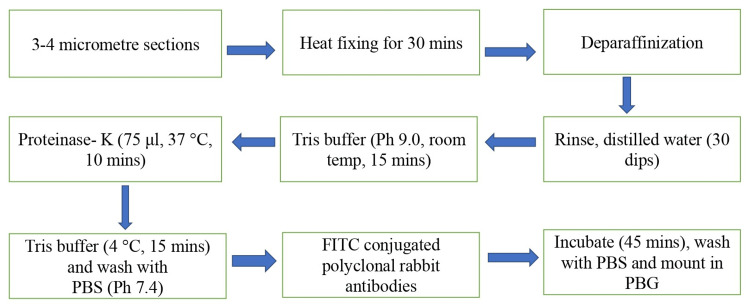
Flowchart depicting the protocol for paraffin-embedded direct immunofluorescence protocol. PBS = phosphate-buffered saline; FITC = fluorescein isothiocyanate; PBG = phosphate-buffered glycerol

Fluorescein isothiocyanate-conjugated polyclonal rabbit antibodies against IgG and IgA were applied, and sections were incubated for 45 minutes, facilitating specific antibody binding. Washing with PBS removed unbound antibodies. Finally, sections were mounted in phosphate-buffered glycerol to preserve the fluorescence signal and protect the slides (Figure 1).

This standardized protocol, based on the existing literature with modifications, aimed to ensure consistent and reliable immunofluorescence staining on paraffin-embedded tissue sections for detecting IgG and IgA in AIBD cases.

Data analysis

Descriptive statistics were used to summarize the demographic and clinical characteristics of the study population. The findings from the DIF analysis were compared with the clinical and histopathological diagnosis to evaluate the concordance and reproducibility of the immunofluorescence staining obtained from the devised protocol.

## Results

Manual PE-DIF was done using a protocol devised from the available literature and subsequent standardization. PE-DIF for IgG and IgA was performed on histopathologically confirmed 15 PV cases, three PF cases, four BP cases, three DH cases, three subcorneal pustular dermatosis (SCPD) cases, one case of linear IgA disease, and one case of pemphigoid gestationis (PG) (Table 1).

**Table 1:** Direct immunofluorescence staining intensities of various autoimmune vesiculobullous lesions. BP = bullous pemphigoid; DH = dermatitis herpetiformis; IgG = immunoglobulin G; PG = pemphigus gestationis; PF = pemphigus foliaceus; PV = pemphigus vulgaris; SCPD = subcorneal pustular dermatosis

Entity-IgG staining	Number of cases with excellent staining	Number of cases with moderate staining	Number of cases with weak staining	Number of cases with no staining	Total number of cases (30)
PV	8	5	2	0	15
PF	2	0	1	0	3
BP	1	0	2	1	4
DH	0	0	0	3	3
SCPD	0	0	1	2	3
Linear IgA	0	0	0	1	1
PG	0	1	0	0	1

**Figure 2:**
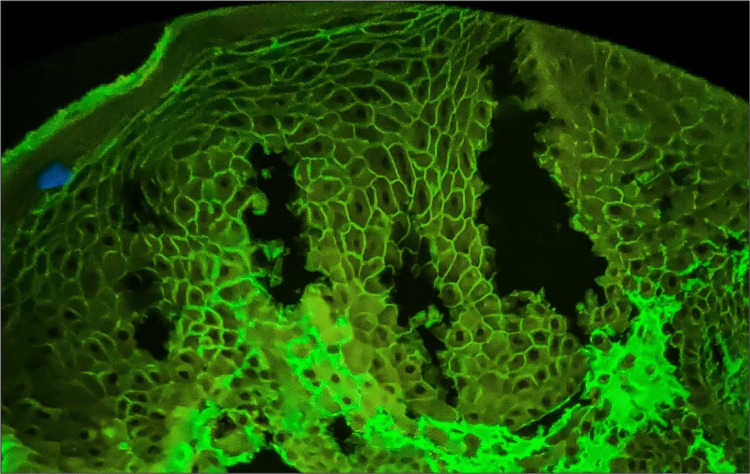
Microphotograph showing DIF positivity for intercellular IgG displaying chicken wire pattern in pemphigus vulgaris. DIF = direct immunofluorescence; IgG = immunoglobulin G

In PV, all 15 cases exhibited a consistent intercellular chicken wire staining pattern for IgG (Figure 2). Eight cases displayed excellent intercellular staining, while five showed moderate staining, and two presented with faint immunofluorescence signals. Background staining correlated with acantholysis severity and inflammatory exudates. Overall, IgG DIF analysis in PV cases demonstrated concordance with expected patterns, reflecting variations in disease activity.

**Figure 3:**
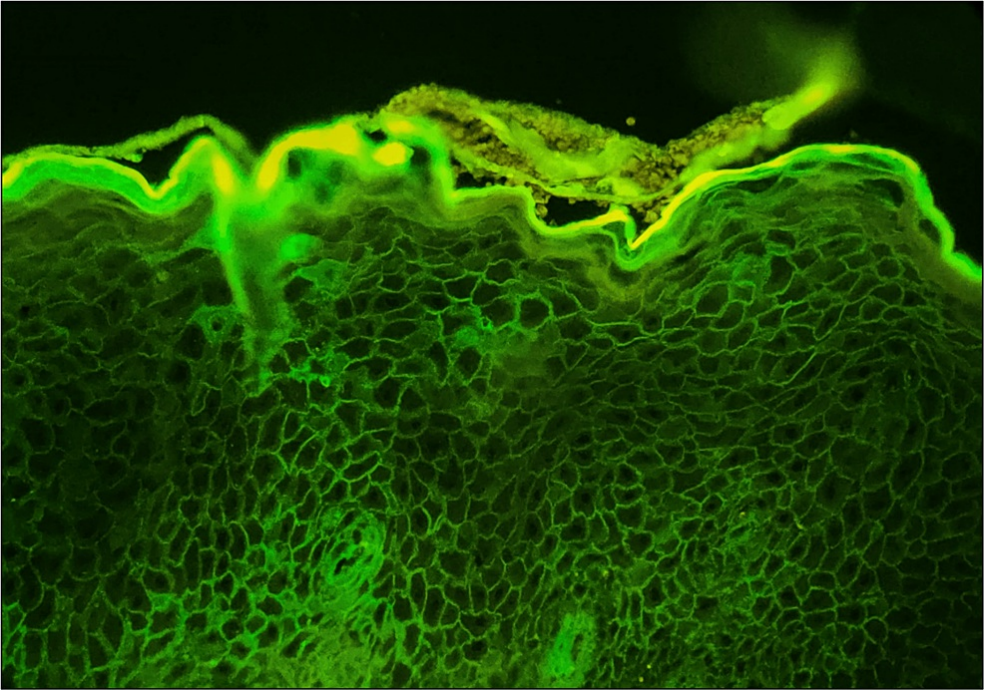
Microphotograph showing DIF positivity for intercellular IgG with chicken wire pattern in pemphigus foliaceous. DIF = direct immunofluorescence; IgG = immunoglobulin G

PF cases (three in total) displayed intercellular IgG staining patterns (Figure 3). Two cases exhibited excellent staining, while one showed faint staining. Staining intensity in PF cases was relatively lesser than in fresh frozen samples, possibly due to the differences in tissue preparation. Despite variations, IgG DIF analysis in PF confirmed consistent intercellular patterns, aligning with expected findings.

**Figure 4:**
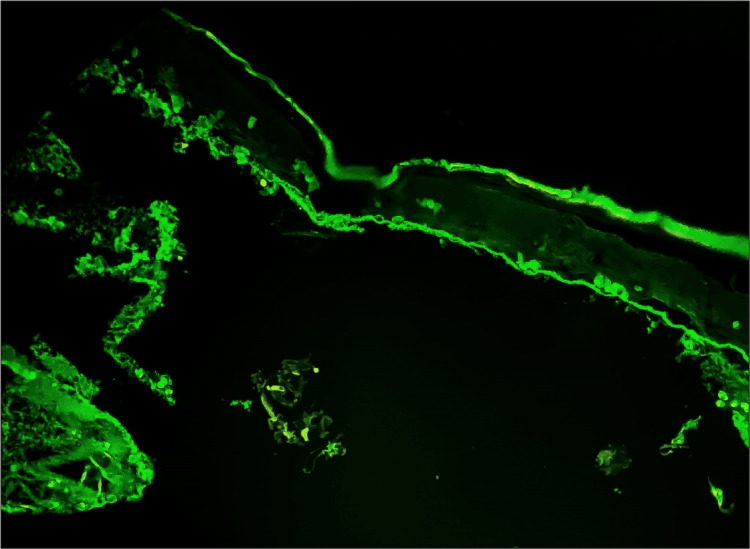
Microphotograph showing linear DIF positivity for IgG along the BMZ of the epidermis in bullous pemphigoid. BMZ = basement membrane zone; DIF = direct immunofluorescence; IgG = immunoglobulin G

BP cases (four in total) demonstrated varied IgG staining patterns (Figure 4). One case showed excellent linear staining along the BMZ, while two displayed moderate-to-faint linear patterns. One case had no detectable staining. Background staining was present in all cases, indicating the need for careful interpretation. Staining variations suggested diverse disease characteristics, emphasizing the importance of considering clinical and histopathological findings for accurate BP diagnosis.

**Figure 5:**
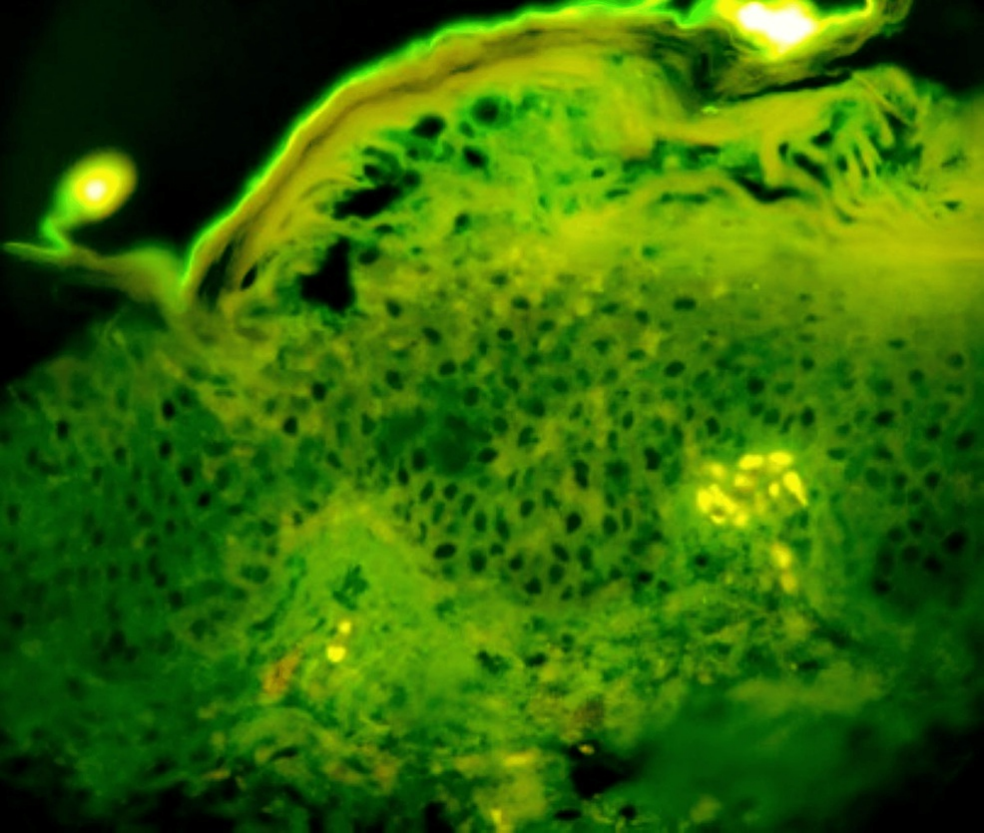
Negative IgG staining in subcorneal pustular dermatosis. IgG = immunoglobulin G

SCPD cases (three in total) showed negative IgG staining, indicating a lack of specific IgG autoantibodies (Figure 5). While this suggests IgG-mediated mechanisms may not play a significant role, other immune mechanisms cannot be ruled out. Comprehensive studies involving multiple immunological markers are warranted for a deeper understanding of SCPD pathogenesis.

**Figure 6:**
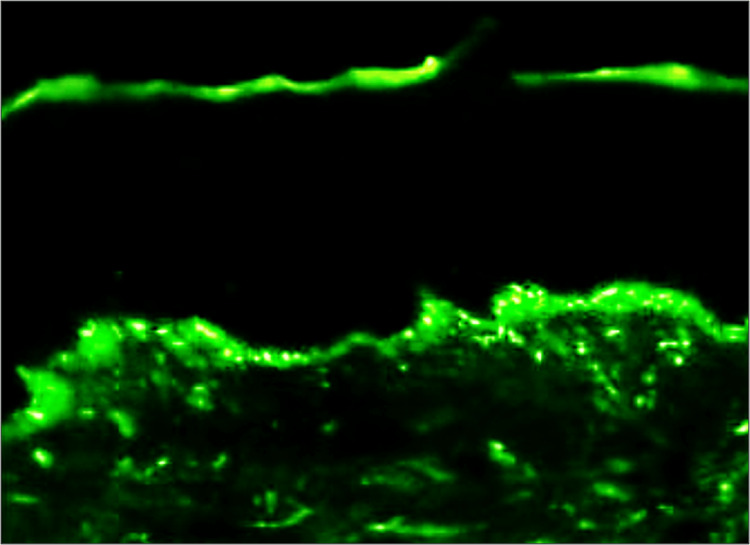
Microphotograph showing granular IgA DIF positivity at the dermo-epidermal junction in case of dermatitis herpetiformis. DIF = direct immunofluorescence; IgA = immunoglobulin A

DH cases (two in total) displayed granular IgA positivity at the dermo-epidermal junction, consistent with characteristic findings (Figure 6). One case did not show any granular IgA positivity due to a technical error. Despite variations in staining patterns between paraffin-embedded and fresh frozen tissue, the presence of IgA at the dermo-epidermal junction confirmed DH diagnosis. Careful interpretation of immunofluorescence results in paraffin-embedded samples is crucial due to tissue preparation and staining technique limitations.

**Figure 7:**
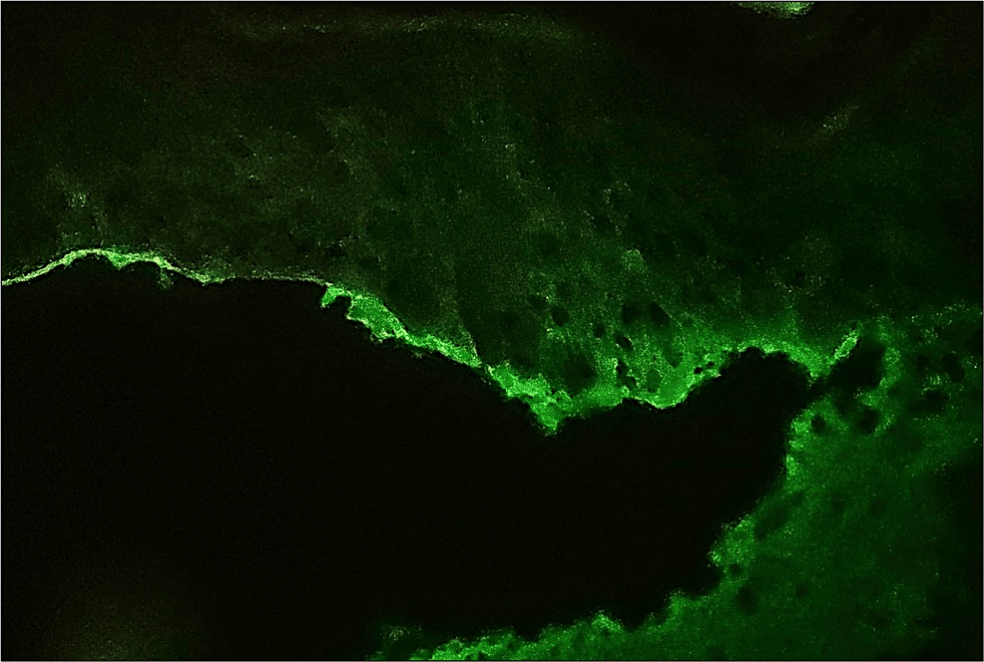
Microphotograph showing DIF positivity of linear IgA deposition along the BMZ in case of linear IgA disease. BMZ = basement membrane zone; DIF = direct immunofluorescence; IgA = immunoglobulin A

The linear IgA disease case exhibited linear IgA positivity along the BMZ, consistent with the expected findings (Figure 7). This pattern differentiated linear IgA disease from other vesiculobullous disorders, highlighting the significance of IgA DIF analysis in diagnosis.

The study noted a rapid reduction in immunofluorescence intensity within 5 to 10 minutes in all cases, suggesting potential instability or degradation of fluorescent antibodies over time. Factors such as enzymatic activity, pH changes, and proteolytic degradation may contribute. Prompt DIF analysis is essential for accurate results, and further optimization may enhance staining stability.

Comparing interpretations with fresh frozen samples revealed similar staining patterns, affirming the feasibility of utilizing paraffin-embedded tissue for DIF analysis.

Several factors may influence DIF diagnostic accuracy for autoimmune vesiculobullous lesions, including biopsy specimen quality, lesion type, disease duration, immune deposit presence, and pathologist experience. Despite higher accuracy in fresh frozen samples, the study supports the usefulness of DIF on paraffin-embedded samples, provided it is performed and interpreted by experienced pathologists.

## Discussion

Autoimmune vesiculobullous lesions arise from an immune-mediated assault on various skin structures, targeting proteins such as Dsg, bullous pemphigoid antigen 1 (BP180), collagen XVII (BP230), and laminin 5 [5]. The resulting autoantibodies can lead to conditions such as pemphigus, bullous pemphigoid, and epidermolysis bullosa acquisita. Diagnosis depends on lesional biopsy for histopathology and perilesional biopsy for DIF. Diagnosis often involves techniques such as DIF, a method utilizing fluorescent-labeled antibodies to detect and localize autoantibodies in skin biopsy samples.

DIF on fresh or frozen tissue is a highly sensitive technique capable of detecting small amounts of autoantibodies. However, it is not without limitations, as it requires specialized storage conditions, is labor-intensive, and is not suitable for archival tissue specimens. This has led researchers to explore the potential of using paraffin-embedded tissue samples for DIF, given their advantages such as archival preservation, wide accessibility, compatibility with various techniques, and cost-effectiveness.

DIF on frozen tissue (DIF-F) can be the method of choice for immune deposits present in the skin and other tissues. DIF can also be performed on formalin-fixed paraffin-embedded tissue (DIF-P) after antigen retrieval with protease [9,10]. Nasr et al. have proven the value of DIF-P in renal pathology [11].

In a study by Singh et al. (2016) involving 215 renal biopsy specimens on which immunofluorescence was performed using paraffin-embedded blocks, immunofluorescence using paraffin-embedded blocks was found to be a useful salvage technique [12]. However, this technique was found to be not without pitfalls, especially due to issues with underdigestion of tissues which may lead to false-positive and false-negative findings.

Valencia-Guerro et al. in 2017 compared the findings of DIF on paraffin-embedded specimens and fresh frozen specimens of 60 skin biopsies and found that DIF on paraffin-embedded blocks was overall less sensitive than on fresh frozen tissue; however, it was found to be a tool with a valuable technique that could aid the diagnosis of vasculitis and immune-bullous and connective tissue disorders when fresh tissue is unavailable [9].

Our study aimed to contribute to this exploration by evaluating the utility of DIF on paraffin-embedded tissue samples for the diagnosis of AIBDs. Rigorous standardization was undertaken to ensure reliable and reproducible results. The findings indicated that DIF can be successfully performed on paraffin-embedded tissue sections, offering a valuable alternative to fresh or frozen samples. Comparing interpretations with fresh frozen samples revealed similar staining patterns, affirming the feasibility of utilizing paraffin-embedded tissue for DIF analysis.

While the advantages of paraffin-embedded tissue samples are evident, our study also identified challenges in immunofluorescence staining. Factors such as inflammatory exudates, prolonged proteinase K digestion, pH variation, and inadequate inactivation of proteinase K influenced background staining. Optimization techniques, including careful selection of tissue samples and standardization of the staining process, were crucial for ensuring reliable and accurate results. Immediate analysis and documentation after staining, along with the use of thin sections, were recommended to minimize unwanted background staining.

Despite the challenges, paraffin-embedded tissue samples offer significant benefits. Archival preservation allows for extended storage periods, essential for retrospective studies, follow-up biopsies, and situations where fresh or frozen samples are unavailable. Their wide accessibility enables collaboration between multiple centers and institutions, fostering comparisons and enhancing research capabilities. Furthermore, paraffin-embedded tissue samples are compatible with various diagnostic techniques, including immunohistochemistry, in situ hybridization, and molecular studies, providing a comprehensive characterization of tissue samples. The cost-effectiveness of using paraffin-embedded tissue samples is particularly valuable in low-resource settings where fresh or frozen samples may be scarce. However, it is crucial to acknowledge that paraffin embedding can affect the antigenicity of autoantigens, potentially leading to decreased sensitivity of DIF. Therefore, optimizing the fixation and embedding process is essential to preserve antigenicity and ensure accurate diagnosis. Additionally, not all paraffin-embedded tissue samples may be suitable for DIF, as some may have degraded or damaged antigens that cannot be detected by this technique. Careful selection of tissue samples is vital for accurate diagnosis.

Our study’s findings align with and complement several other investigations into the diagnostic accuracy of DIF on paraffin-embedded skin biopsy samples for AIBDs.

A study by van Beek et al. (2017) regarding the performance of DIF on paraffin-embedded skin biopsy samples for the diagnosis of epidermolysis bullosa acquisita demonstrated sensitivity and specificity of 96% and 100%, respectively [13]. This study further emphasizes the efficacy of DIF on paraffin-embedded tissue samples in identifying autoantibodies targeting specific proteins associated with epidermolysis bullosa acquisita. Collectively, these studies, along with our findings, contribute to the growing body of evidence supporting the use of DIF on paraffin-embedded skin biopsy samples as a reliable and accurate diagnostic tool for AIBDs.

In a study by Abreu-Velez et al. (2018) focusing on pemphigus, the reported sensitivity and specificity of DIF on paraffin-embedded skin biopsy samples were 88% and 95%, respectively, supporting the utility of paraffin-embedded tissue for pemphigus diagnosis [14]. These findings underscore the high diagnostic accuracy of DIF on paraffin-embedded tissue in detecting autoantibodies associated with BP.

The consistently high sensitivity and specificity reported in multiple studies underscore the potential of this technique to effectively detect autoantibodies and aid in the diagnosis of these complex dermatological conditions. By utilizing paraffin-embedded tissue samples, clinicians can overcome the limitations associated with fresh or frozen samples, including limited availability and the need for specialized storage conditions. Despite challenges in immunofluorescence staining, careful optimization ensures accurate and reproducible results, making DIF on paraffin-embedded tissue a valuable tool in the diagnostic armamentarium for AIBDs.

Study limitations

The small sample size included in the study may not be truly representative of the population. Although utmost care was taken to avoid selection bias, a certain level of unavoidable selection bias might have occurred as the study was done in a tertiary care center. Even though most of the common disease entities of AIBDs were included in the study, rare entities such as paraneoplastic pemphigus, epidermolysis bullosa, and cicatricial pemphigoid were not included. This requires further studies with extensive inclusion of all these entities.

## Conclusions

This research seeks to establish paraffin-embedded specimens as a convenient alternative to fresh tissue for DIF analysis, potentially revolutionizing diagnostic accessibility and patient care in AIBDs and the high diagnostic accuracy with a sensitivity and specificity of 94% and 97%, respectively. These findings, consistent with prior research, highlight the valuable role of DIF on paraffin-embedded tissue as an adjunctive diagnostic tool, particularly in cases lacking fresh tissue or inconclusive results from other modalities. The clinical implications are substantial, suggesting clinicians consider this technique in their diagnostic approach. Future research should prioritize larger, multicenter cohorts to validate our findings and assess the clinical utility of DIF on paraffin-embedded tissue in treatment decisions and predicting disease outcomes.
